# Liver biochemistry and associations with alcohol intake, hepatitis B virus infection and Inuit ethnicity: a population-based comparative epidemiological survey in Greenland and Denmark

**DOI:** 10.3402/ijch.v75.29528

**Published:** 2016-02-26

**Authors:** Karsten Fleischer Rex, Henrik Bygum Krarup, Peter Laurberg, Stig Andersen

**Affiliations:** 1 Department of Internal Medicine, Queen Ingrid's Hospital, Nuuk, Greenland; 2 Arctic Health Research Centre, Department of Clinical Medicine, Aalborg University Hospital, Aalborg, Denmark; 3 Department of Clinical Biochemistry, Aalborg University Hospital, Aalborg, Denmark; 4 Department of Endocrinology, Aalborg University Hospital, Aalborg, Denmark; 5 Department of Geriatric Medicine, Aalborg University Hospital, Aalborg, Denmark

**Keywords:** alcohol intake, hepatitis B virus infection, liver biochemistry, ethnicity Greenland Inuit, migration, Denmark Arctic Greenland

## Abstract

**Background:**

Hepatitis B virus (HBV) infection is common in Arctic populations and high alcohol intake has been associated with an increased risk of a number of diseases. Yet, a description of the influence of alcohol intake in persons with HBV infection on liver biochemistry is lacking.

**Objective:**

We aimed to describe the association between reported alcohol intake and liver biochemistry taking into account also HBV infection, ethnicity, Inuit diet, body mass index (BMI), gender and age in an Arctic population.

**Design and methods:**

Population-based investigation of Inuit (n=441) and non-Inuit (94) in Greenland and Inuit living in Denmark (n=136). Participants filled in a questionnaire on alcohol intake and other life style factors. Blood samples were tested for aspartate aminotransferase (AST), gamma-glutamyl transferase (GGT), alkaline phosphatase (ALP), bilirubin, albumin, hepatitis B surface antigen, hepatitis B surface antibody and hepatitis B core antibody. We also performed physical examinations.

**Results:**

Participation rate was 95% in Greenland and 52% in Denmark. An alcohol intake above the recommended level was reported by 12.9% of non-Inuit in Greenland, 9.1% of Inuit in East Greenland, 6.1% of Inuit migrants and 3.4% of Inuit in the capital of Greenland (p=0.035). Alcohol intake was associated with AST (p<0.001) and GGT (p=0.001), and HBV infection was associated with ALP (p=0.001) but not with AST, GGT, bilirubin or albumin in the adjusted analysis. Inuit had higher AST (p<0.001), GGT (p<0.001) and ALP (p=0.001) values than non-Inuit after adjustment for alcohol, diet, BMI and HBV exposure. Ethnic origin modified the association between alcohol and AST, while HBV infection did not modify the associations between alcohol and liver biochemistry.

**Conclusions:**

Non-Inuit in Greenland reported a higher alcohol intake than Inuit. Ethnic origin was more markedly associated with liver biochemistry than was alcohol intake, and Greenlandic ethnicity modified the effect of alcohol intake on AST. HBV infection was slightly associated with ALP but not with other liver biochemistry parameters.

The many effects of alcohol (ethanol) consumption on the human body and mind are well understood. A high consumption of alcohol has been described in societies going through rapid transitions and societies in the circumpolar region are no exception (1–4). A high alcohol consumption has been associated with an increased risk of a number of diseases (5–8), accidents (9), violence (10), sexual assaults (11) and suicides (12), all of which lead to increased mortality (3,6,9,10,13–17). However, limited research has focused on alcohol consumption and liver disease in Inuit.

Hospital discharge diagnoses related to liver disease have not been found to differ between Inuit (ethnic Greenlanders) and non-Inuit with high alcohol consumption (18). On the other hand, liver biochemistry has been found to be less affected in Inuit compared to non-Inuit attending alcohol treatment centres in Greenland or Denmark (19). Still, the prevalence of alcohol-induced liver disease parallels the alcohol consumption in other populations (20) and there is a distinct relationship between alcohol consumption and liver cirrhosis (21).

The risk of liver damage induced by alcohol may increase when other liver injuries are present (22), and coexisting viral hepatitis may influence the consequences of alcohol consumption (23). Hepatitis B virus (HBV) infection has been shown to be endemic among the Indigenous peoples of the Arctic with very high prevalence rates among Inuit (24–27). Still, the influence of combined alcohol and HBV infection on liver biochemistry in Inuit remains unsettled.

This led us to investigate Inuit and non-Inuit in Greenland and Inuit in Denmark for the association between alcohol intake and liver biochemistry, also taking into account HBV infection.

## Methods

Greenland has a population of around 57,000 people mostly of Inuit (Eskimo) descent. Of the 7,000 people born outside Greenland, most were born in Denmark. Around 18,000 people in Denmark are of Greenlandic descent, and around 5,000 people living in Denmark were born in Greenland (28,29).

The cultural tradition of the people of Greenland is to call themselves Inuit (humans); the term “Eskimo” is considered inappropriate and is seldom used, although other words exist in the native tongue to address the population or parts of the native population according to geography (Tunumiut, Avannaamiut, Kujataamiut, Kitaamiut, Kalaallit and so on). Inuit also refers descendants of the Greenlandic Indigenous people. For the purpose of ethnicity, in our study, we chose to define an Inuit as an individual born in Greenland with at least one parent born in Greenland.

The methods used in the survey in Greenland and Denmark have been described in detail previously (25,26,30,31). In short, participants’ names and addresses were obtained from the National Civil Registration System in which every person living in Denmark, the Faeroe Islands and Greenland is recorded. We selected a random sample of 25% of men and women aged 50 through 69 years in Nuuk in West Greenland (total population 12,909; in the age group, 1,920; 75% Inuit) and all men and women in that age range in Tasiilaq (total population 1,724; in the selected age group 197, 95% Inuit) and the settlements of Tiniteqilaaq, Sermiligaaq, Kuummiut and Kulusuk (1,093; 161; 98% Inuit) in East Greenland. A letter of invitation was delivered to each subject by the local hospital porter or the nursing station attendant. Non-responders were invited 3 times. The investigation took place at the local hospital or nursing station or, by request, in home visits. Data were collected during late summer and autumn 1998. The participation rate was 95%. The initial selection of participants in Nuuk was based on the hospital registrations system. This lacked updating, and the names and addresses were subsequently validated with the National Civil Registration System.

**Fig. 1:**
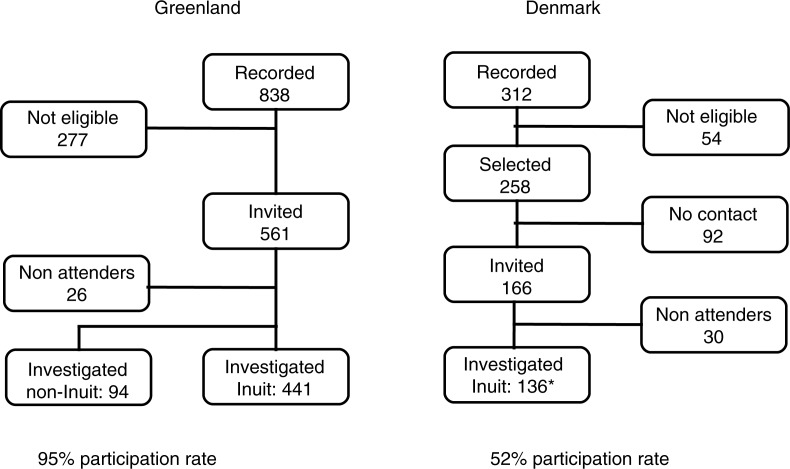
Flow chart for inclusion of Inuit and non-Inuit in Greenland and Inuit in Denmark. The study population comprised 577 Inuit living in Greenland or Denmark and 94 non-Inuit in Greenland aged 40–69 years, 1998 and 2006. *Of Inuit in Denmark 37 were aged 40 through 49 years.

For the investigation in Denmark, we invited all Inuit migrants from Greenland to Denmark aged 40 through 69 years recorded and now living in the city of Aarhus or Aalborg. The lowering of the age limit for inclusion from 50 to 40 years in Denmark was due to the limited number of Inuit in the age group that was included in Greenland. A letter of invitation was sent by mail to all 312 subjects identified in Denmark (Fig. 1). As in Greenland, non-responders were invited 3 times. Each of the 220 responders was contacted by telephone for a short telephone interview to clarify whether they had Inuit ancestry. If they were of Inuit origin, they were invited in for interviews and procedures similar to those carried out among the participants in Greenland. The investigation took place at the local hospital in 2006. None of the participants were reimbursed for their time.

Figure 1 is a flow chart of selection and inclusion of participants. In Denmark, 166 were invited to participate and 30 refrained from attending. Fifty-four did not have parents born in Greenland, had died, moved out of the study area, or were unable to give informed consent for medical reasons. We were unable to determine the eligibility of the 92 subjects in Denmark who did not respond (Fig. 1). In Greenland, 277 had died or moved but were still registered at the address due to late reporting. Twenty-six did not attend the investigation.

The regional ethics committee for Viborg and Nordjylland County approved the study in Denmark (VN-20060038). The Commission for Scientific Research in Greenland approved the study in Greenland (505-99). A letter of invitation was sent to all selected subjects and all participants gave informed written consent in Danish or Greenlandic by participant choice.

### Investigational procedures

Information on parents’ birthplace, smoking habits, alcohol intake and dietary habits was obtained by using an interview-based questionnaire in Greenlandic or Danish, as appropriate for the subject. Questions were asked as written in the questionnaires. Twelve grams of alcohol was the equivalent of 1 unit. Average alcohol intake above 2 units per day for women and 3 for men defined an intake above the recommended levels (32). Dietary habits were categorized based on the number of days per week with the main meal of traditional Greenlandic food items (that is, Greenlandic seal, whale, wild fowl, fish, reindeer, musk ox and hare). Participants were classified based on the number of days per week that the main meal consisted of Greenlandic food items. The dietary assessment method has been described in detail previously and validated in Greenland Inuit (31,33). Information on gender and age was obtained from the National Civil Registration System. A physical examination was performed and included recording height and weight in indoor clothing, disabilities, scleral jaundice, spider naevi and signs of hepatic decompensation such as confusion, jaundice, fluid retention and cachexia. Body mass index (BMI) was calculated as weight in kilograms divided by height in metres squared.

A venous blood sample was drawn using minimal tourniquet, separated and stored at −20 C. Blood samples were blinded using an 8-digit code, and analysed in random order. Blood sampling was omitted for 4 participants in Denmark and 1 in Greenland, in compliance with the participants’ choice.

The physical examination and venepuncture were performed by KFR or SA in Denmark and SA or PL in Greenland.

### Serology

Testing for hepatitis B surface antigen (HBsAg), hepatitis B surface antibody (anti-HBs) and hepatitis B core antibody (anti-HBc-total) was performed using HBsAg (V2), confirmatory HBsAg, AUSAB^®^, CORE™ (Abbott Axsym™ System, Abbott Diagnostics a/s, Germany) (25,26). Participants were classified as never exposed if all markers were negative, previously exposed if anti-HBs or anti-HBc-total were positive and as currently infected if HBsAg was positive. This classification was used in the statistical analysis.

### Biochemistry

Aspartate aminotransferase (AST), gamma glutamyltransferase (GGT), bilirubin, alkaline phosphatase (ALP) and albumin were measured on a Vitros Chemistry System 950 (Ortho-Clinical Diagnostics, Inc., Raritan, NJ, USA). Alanine aminotransferase and INR were not measured as serum was frozen for transport to the laboratory in Denmark which decreases the reproducibility (34,35). Only data from Inuit and non-Inuit in Greenland were used for comparisons and used in figures and tables, as these serum samples were analysed in the same assay runs in random order.

### Statistics

**Fig. 2:**
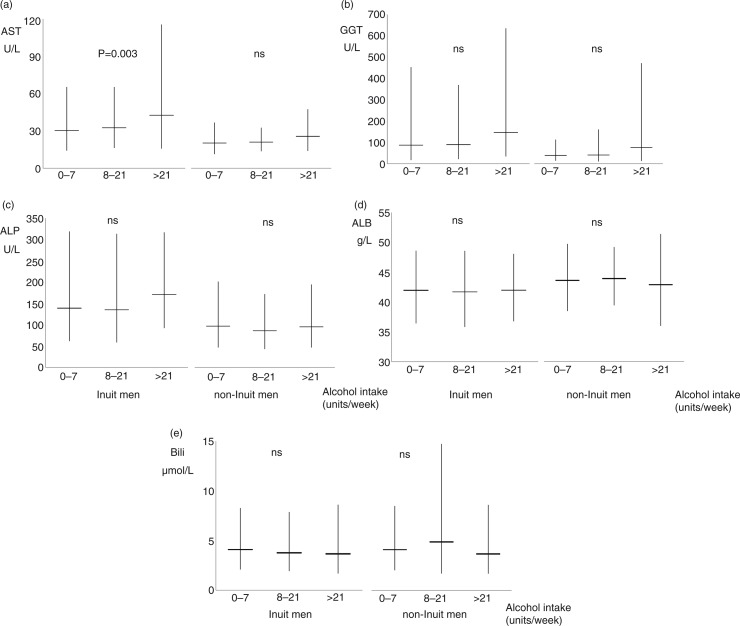
(a) Aspartate aminotransferase (AST), (b) gamma glutamyltransferase (GGT), (c) alkaline phosphatase (ALP), (d) albumin, and (e) bilirubin in 252 Inuit (left) and 75 non-Inuit (right) men in Greenland by alcohol intake groups. Mean values are shown with 95% confidence intervals. P-values are for trends with increasing alcohol consumption. ns (non-significant) and U/L designate P>0.1 and Units/Litre.

**Fig. 3:**
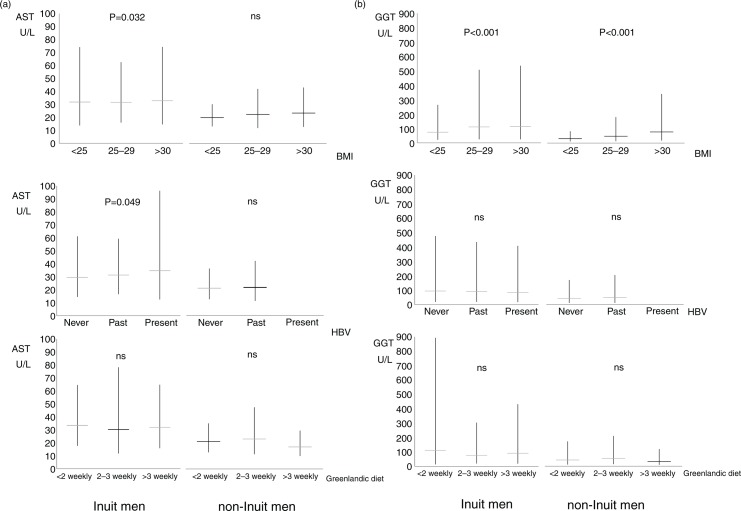
(a) Aspartate aminotransferase (AST) and (b) gamma glutamyltransferase (GGT) in 252 Inuit (left) and 75 non-Inuit (right) men in Greenland. Levels are given with BMI (upper panel), HBV exposure (middle panel) and frequency of intake of Inuit diet (lower panel). HBV exposure was classified as never exposed if all markers were negative, previously exposed if anti-HBs or anti-HBc were positive, and presently infected if HBsAg was positive. Mean values are shown with 95% confidence intervals. P-values are for trends with increasing BMI, HBV exposure or Greenlandic diet. Ns (non-significant) and U/L designate P>0.1 and Units/Litre.

Frequencies are given as mean, with 95% confidence intervals or as median values with 25 and 75 percentiles, as appropriate. Mean values for Figs. 2 and 3 were calculated on ln-transformed data that were subsequently transformed back. A chi-square test was used for a comparison of proportions, a Mann-Whitney U-test and Kruskall-Wallis test for a comparison of median values, and Kendall's tau was used to test for trends.

Liver function tests were entered as dependent variables in multivariate linear regression models after logarithmic transformation. Age (years, included as a continuous variable), gender (men or women), origin (Inuit or non-Inuit), BMI (kg/m^2^, continuous variable), intake of alcohol (using 5 categories, see Table I), Greenlandic diet (days/week, included as a continuous variable) and HBV infection (HBsAg positivity, yes or no) were entered as explanatory variables. Liver function tests above the upper reference limit were entered as dependent variables in logistic regression models. The effect modification of HBV infection and ethnic origin were tested as interaction terms in the logistic regression models. Data were processed and analysed using Corel Quattro Pro X3 (Corel Corporation, Ottawa, Ontario, Canada) and the Statistical Package for the Social Sciences version 13.0 (SPSS Inc., Chicago, Ill., USA). A 2-sided p-value of less than 0.05 was considered significant in the statistical analyses.

**Table I:** Characteristics of 634 study participants living in Greenland and Denmark, 1998 and 2006

	Inuit n (%)	Non-Inuit n (%)	Both n (%)	p^a^
All	540 (85.2)	94 (14.8)	634 (100)	
Investigated				<0.001
West Greenland	153 (28.3)	59 (62.8)	212 (33.4)	
East Greenland	288 (53.3)	35 (37.2)	323 (51.0)	
Denmark	99 (18.3)		99 (15.6)	
Gender				<0.001
Men	252 (46.7)	75 (79.8)	327 (51.6)	
Women	288 (53.3)	19 (20.2)	307 (48.4)	
Age				<0.001
< 60 years	337 (62.4)	79 (84.0)	416 (65.6)	
60+ years	203 (37.6)	15 (16.0)	218 (34.4)	
Smoker^b^				<0.001
Never	67 (12.5)	25 (26.6)	92 (14.6)	
Past	74 (13.7)	17 (18.1)	91 (14.4)	
Present	396 (73.7)	52 (55.3)	448 (71.0)	
Alcohol intake (units/week)^c^^d^				<0.001
Never	158 (29.9)	9 (9.7)	167 (26.8)	
1–7	191 (36.1)	42 (45.2)	233 (37.5)	
8–14	111 (21.0)	24 (25.8)	135 (21.7)	
15–21	44 (8.3)	6 (6.5)	50 (8.0)	
21 +	25 (4.7)	12 (12.9)	37 (5.9)	
Alcohol above recommended^d^^e^				0.061
Yes	38 (7.2)	12 (12.9)	50 (8.0)	
No	491 (92.8)	81 (86.7)	572 (92.0)	
Traditional Inuit foods^f^				<0.001
< 2 weekly	104 (19.6)	70 (75.3)	174 (27.9)	
2–3 weekly	99 (18.6)	19 (20.4)	118 (18.9)	
> 3 weekly	328 (61.8)	4 (4.3)	332 (53.2)	
Hepatitis B virus^g^^h^				<0.001
Never	147 (27.5)	70 (75.3)	217 (34.6)	
Past	295 (55.2)	23 (24.7)	318 (50.7)	
Present	92 (17.2)	0 (0.0)	92 (14.7)	
BMI^i^				ns
< 30 kg/m^2^	394 (80.9)	74 (81.3)	468 (81.0)	
30+ kg/m^2^	93 (19.1)	17 (18.7)	110 (19.0)	

Inuit and non-Inuit aged 50–69 years. ns, non-significant (p>0.1).

a Chi-square test;

b 3 missing;

c one unit contains 12 g ethanol;

d 12 missing;

e weekly intake of more than 14 units for women and 21 units for men;

f 10 missing;

g present: HBsAg positive, previous: anti-HBs or anti-HBc were positive, never: if all markers were negative;

h 7 missing;

i 56 missing.

## Results

The participation rates were 95% in Greenland and 52% in Denmark. All participants in Denmark were Inuit according to the inclusion criteria, while 94 participants in Greenland were non-Inuit.

The flow chart for inclusion of participants in both Greenland and Denmark is shown in Fig. 1.

Participant characteristics are listed in Table I. Gender distribution differed between participants in Greenland and Denmark (58% men in Greenland; 76% women in Denmark, p<0.001). More men than women had current HBV infection (men 17%, women 10%; p=0.01), and men had a higher alcohol intake than women (p<0.001). Isolated anti-HBs was seen in 39 individuals. This group included 5 non-Inuit.

Table II shows the characteristics of participants by alcohol intake groups. An alcohol intake above the recommended level was more frequent in non-Inuit men in Greenland than in any Inuit group (p=0.051). The alcohol intake among Inuit was highest in East Greenland, followed by Inuit in Denmark, with the lowest intake in the capital city in West Greenland. In general, younger, non-Inuit, smoking men had the highest alcohol intake.

**Table II:** Characteristics by alcohol intake of 540 Inuit in Greenland and Denmark and 94 Non-Inuit in Greenland aged 50–69 years, 1998 and 2006

	Units of alcohol weekly					
				
	7 or less n (%)	8 through 21 n (%)	More than 21 n (%)	Above recommended^a^ n (%)	p^b^	p^c^
All participants					0.004	0.051
Inuit in East Greenland	185 (64.7)	84 (29.4)	17 (5.9)	26 (9.1)		
Inuit in West Greenland	94 (63.9)	51 (34.7)	2 (1.4)	5 (3.4)		
Inuit in Denmark	70 (72.9)	20 (20.8)	6 (6.3)	7 (7.3)		
Non-Inuit in Greenland	51 (54.8)	30 (32.3)	12 (12.9)	12 (12.9)		
Ethnicity					<0.001	0.061
Inuit	349 (66.0)	155 (29.3)	25 (4.7)	38 (7.2)		
Non-Inuit	51 (54.8)	30 (32.3)	12 (12.9)	12 (12.9)		
Gender					<0.001	ns
Men	177 (54.6)	119 (36.7)	28 (8.6)	28 (8.6)		
Women	223 (74.8)	66 (22.1)	9 (3.0)	22 (7.4)		
Age					<0.001	0.011
< 60 years	241 (59.1)	137 (33.6)	30 (7.4)	41 (10.0)		
60+ years	159 (74.3)	48 (22.4)	7 (3.3)	9 (4.2)		
HBV exposure					ns	0.006
Never	133 (63.0)	66 (31.3)	12 (5.7)	13 (6.2)		
Past	206 (65.8)	93 (29.7)	14 (4.5)	22 (7.0)		
Present	56 (61.5)	24 (26.4)	11 (12.1)	15 (16.5)		
Smoker					<0.001	<0.001
Never	68 (74.7)	22 (24.2)	1 (1.1)	2 (2.2)		
Past	74 (83.1)	15 (16.9)	0 (0.0)	1 (1.1)		
Present	257 (58.4)	147 (33.4)	36 (8.2)	47 (10.7)		
BMI					0.013	ns
<30	284 (61.9)	144 (31.4)	31 (6.8)	39 (8.5)		
30 +	79 (73.8)	23 (21.5)	5 (4.7)	8 (7.5)		

One unit of alcohol contains 12 g of ethanol. ns, non-significant (p>0.1).

a A weekly intake of more than 14 units of ethanol for women and 21 units for men;

b Kendall's tau for difference with increasing alcohol intake;

c chi-square test for difference between those with an alcohol intake below/above the recommended maximum.

Table III shows measures of body build by alcohol intake groups. Height, weight and BMI did not differ between alcohol intake groups, except for a slight decrease in weight and BMI with increasing alcohol consumption in Inuit men.

**Table III:** Physical findings by alcohol intake among 577 Inuit in Greenland and Denmark and 94 Non-Inuit in Greenland aged 50–69 years (Inuit in Denmark aged 40–69 years), 1998 and 2006

	Units of alcohol weekly			Above recommended^a^		
				
	7 or less	8 thru 21	More than 21		p^b^	p^c^
Height (cm)						
Men						
Inuit	164 (160; 169)	164 (160; 169)	166 (162; 171)	166 (162; 171)	ns	ns
Non-Inuit	177 (170; 180)	178 (170; 182)	173 (172; 177)	173 (172; 177)	ns	ns
Women						
Inuit	155 (150; 159)	155 (150; 159)	155 (152; 160)	154 (150; 159)	ns	ns
Non-Inuit	167 (162; 178)	161 (158; 173)	na	na	ns	na
Weight (kg)						
Men						
Inuit	70 (62; 77)	65 (60; 72)	64 (56; 83)	64 (56; 83)	0.068	ns
Non-Inuit	80 (73; 93)	78 (70; 86)	85 (72; 90)	85 (72; 90)	ns	ns
Women						
Inuit	59 (50; 74)	58 (49; 65)	53 (45; 62)	56 (49; 67)	ns	ns
Non-Inuit	68 (60; 83)	75 (57; 80)	na	na	ns	na
BMI (kg/m^2^)						
Men						
Inuit	25.7 (22.7; 28.7)	24.0 (22.5; 27.1)	23.4 (20.8; 27.7)	23.4 (20.8; 27.7)	0.036	ns
Non-Inuit	26.4 (24.2; 29.3)	25.1 (22.7; 27.8)	27.2 (23.5; 29.7)	27.2 (23.5; 29.7)	ns	ns
Women						
Inuit	23.9 (21.1; 30.0)	23.0 (20.7; 27.4)	22.5 (18.7; 25.0)	23.2 (19.6; 27.8)	ns	ns
Non-Inuit	22.4 (21.2; 28.6)	26.3 (22.1; 30.1)	na		ns	na

Median values (25-; 75-percentiles). One unit of alcohol contains 12 g of ethanol. ns, non-significant (p>0.1); na, not applicable.

a Weekly intake of more than 14 units for women and 21 units for men;

b Kendall's tau for difference with increasing alcohol intake;

c Mann-Whitney U-test for difference between those with an alcohol intake below/above the recommended maximum.

Figure 2 shows liver biochemistry in Inuit and non-Inuit men for 3 alcohol intake categories. AST (panel A), GGT (panel B) and ALP (panel C) were higher in Inuit than in non-Inuit men irrespective of alcohol intake while differences were smaller for albumin (panel D) and bilirubin (panel E). Inuit and non-Inuit men showed similar trends in liver biochemistry with increasing alcohol intake but only the increase in AST with higher alcohol intake in Inuit was statistically significant (p=0.003). Similar patterns were seen for women (not shown in figures).

Alcohol intake questions in Greenland were blank in 12 participants (hereof 3 men, 1 non-Inuit). This group had a median GGT of 135 U/L compared to the overall medians of 55, 72 and 103 U/L in the groups with a weekly alcohol consumption of 7 units or less, 8 through 20 units, and 21 or more units, respectively.

Figure 3 depicts the associations between BMI (top panel), HBV status (central panel) or traditional Inuit diet (lower panel), and AST (Fig. 3a) or GGT (Fig. 3b). Again, GGT and AST were higher in Inuit than in non-Inuit men. GGT increased with higher BMI (p<0.001) but did not differ with HBV status or diet (Fig. 3b). For Inuit men, AST increased slightly with increasing BMI and having been exposed to HBV (Fig. 3a).

Table IV shows factors important to liver biochemistry as evaluated in multivariate regression models. Alcohol consumption and ethnic origin were associated with AST, GGT and ALP.

**Table IV:** Factors associated with liver biochemistry in multivariate analyses among 441 Inuit and 94 Non-Inuit in Greenland aged 50–69 years in 1998

	AST		GGT		ALP		Bilirubin		Albumin	
					
	Beta	p	Beta	p	Beta	p	Beta	p	Beta	p
Age	0.00	ns	−0.00	ns	−0.05	ns	0.01	0.007	−0.00	ns
Gender	−0.07	0.04	−0.15	0.054	−0.03	ns	−0.11	0.002	0.00	ns
BMI	0.22	0.013	1.19	<0.001	0.02	ns	0.07	ns	0.05	0.007
Ethnic origin	−0.43	<0.001	−0.83	<0.001	−0.22	0.001	0.06	ns	0.01	ns
Alcohol		0.001		<0.001		0.001		ns		ns
Never		reference		reference		reference		reference		reference
0–7 units/week	0.02	ns	0.02	ns	−0.16	<0.001	−0.02	ns	0.02	ns
8–14 units/week	0.12	0.024	0.27	0.004	−0.11	0.025	0.01	ns	0.00	ns
15–21 units/week	0.10	ns	0.09	ns	−0.17	0.009	0.06	ns	0.02	ns
>21 units/week	0.31	<0.001	0.77	<0.001	−0.02	ns	0.10	ns	0.00	ns
Greenlandic diet	0.01	ns	0.03	ns	0.05	<0.001	0.00	ns	−0.01	<0.001
HBsAg positive	0.03	ns	−0.12	ns	0.17	0.001	0.08	ns	−0.01	ns

AST, aspartate aminotransferase; GGT, gammaglutamyl transferase; ALP, alkaline phosphatase. Dependent variables entered in multivariate linear regression models were AST, GGT, ALP, bilirubin and albumin after ln-transformation. Explanatory variables entered were advancing age (year), women/men (reference), increasing lnBMI (kg/m^2^), ethnic origin (Inuit (reference)/non-Inuit), alcohol intake (no intake reference), intake of traditional Inuit diet (days/week, no intake reference) and HBsAg positive (yes/no (reference)). Only data from Greenland were included as these were analysed mixed in random order. ns, non-significant (p>0.1).

Determinants of GGT above the upper reference limit (73 U/L for men and 43 U/L for women) were ethnic origin (p<0.001; odds ratio (OR), 95% confidence interval (CI): 8.8, 3.8–21 (non-Inuit reference)), BMI (p<0.001; OR, 95%-CI; 3.4, 2.0–5.8 (BMI<30 kg/m^2^ reference)) and alcohol intake above the recommended level (p=0.003; OR, 95%-CI; 3.1, 1.5–6.6 (never users reference) in multivariate logistic regression models. Similarly, AST above 50 U/L for men and 35 U/L for women was independent of HBV status (p=0.57) and diet (p=0.91) but related to alcohol (p=0.038; OR, 95%-CI: 2.6, 1.1–6.2), ethnic origin (p=0.016; OR, 95%-CI: 6.1, 1.4–26) and gender (p=0.007; OR, 95%-CI: 2.3, 1.3–4.2 (women reference)) in the adjusted analysis. ALP, bilirubin and albumin were not associated with any of the parameters in multivariate logistic regression models.

The influence of alcohol on liver biochemistry was not modified by HBV infection when evaluated as an interaction term in regression models. Ethnic origin modified the association between alcohol and AST (interaction term, p=0.024), but not the association with any of the other liver parameters.

## Discussion

Reported alcohol consumption was lower among Inuit in Greenland and Denmark than in non-Inuit in Greenland. Alcohol intake was associated with AST, GGT and ALP but not bilirubin, albumin or measures of body build. HBV infection did not modify the association between alcohol intake and liver function tests, while ethnic origin modified the relation between alcohol and AST. Interestingly, Greenland Inuit had higher levels of AST, GGT and ALP than non-Inuit, regardless of alcohol intake.

Transitions in a society contribute to population stress that may herald an excess alcohol intake (36). Major changes in Eastern Europe during a transition of political and economic changes were accompanied by a marked increase in alcohol intake (37). Greater acculturation in Inuit societies has been suggested as a transition that contributes to the higher use of alcohol to cope with stress (38). The transition of society in the capital city Nuuk started more than 50 years ago and hence Inuit in Nuuk may have become more accustomed to changes. This may explain our finding of a lower alcohol intake in the capital city Nuuk, as well as among younger age groups (39). Transition of the society in East Greenland is delayed compared to the capital city Nuuk. This may contribute to the higher alcohol consumption found in rural East Greenland compared to Nuuk in our study.

A high alcohol consumption among Inuit has been reported previously (1,3,4,40) with lower intake levels in younger individuals (39). In our study, 29% of Inuit reported being abstainers compared to 9.7% of non-Inuit. We found a lower frequency of high alcohol intake among Inuit in Nuuk, which may be related to several campaigns that featured data from the vast research on the adverse health consequences of high alcohol consumption. Also, the capital city, with a population 10 times that of the town Tasiilaq and around 100 times that of a small settlement, provides more employment opportunities, treatment choices, leisure activities and a diversity of people that makes it easier to merge with groups in the society with a lower alcohol intake. This provides opportunities to support reduced alcohol consumption.

Ethnic differences exist in the alcohol-metabolizing enzymes (41), and alcohol metabolism varies across Asian ethnicities (42). Inuit is a distinct ethnic group with Asian ancestry and a difference in alcohol metabolism, as is seen between Asian populations has been suggested (3). It has been suggested that an altered conversion rate of alcohol that leads to the excess build-up of acetaldehyde, and hence a more severe response to alcohol, could reduce heavy alcohol use and related diseases (42). However, Bjerregaard and colleagues did not confirm the presence of the Asian genotype pattern in a study on the genetic variation in alcohol-metabolizing enzymes among Inuit (43). Yet, AST may reflect alcohol consumption (41,44) and we found an increase in AST with increased reported alcohol consumption. Also, we found a higher AST level in Inuit compared to non-Inuit, even though they reported lower alcohol consumption than non-Inuit. This was the case even among abstainers for whom AST was higher in Inuit compared to non-Inuit. Furthermore, ethnic origin modified the association between alcohol and AST. These findings suggest ethnic differences in liver biochemistry irrespective of alcohol consumption. The findings are interesting, although residual confounding cannot be ruled out.

Additional causes for hepatic injury may explain why some people who abuse alcohol are more likely than others to develop liver disease. It has been suggested that both HBV and hepatitis C virus infection influence the course of alcohol-related liver disease (23) and more severe liver disease has been reported in patients with HBV infection compared to those without (45). HBV is endemic in Arctic populations, and we previously reported HBsAg positivity in 29% of East Greenlanders (25,26). Still, we found no association between HBV infection and AST or GGT in Inuit. Nor did HBV infection modify the effect of alcohol on liver biochemistry. This may be attributable to differences in either drinking patterns, the metabolism of alcohol in Inuit or the subtype of HBV (25).

Hepatitis C and D virus infection may influence liver biochemistry, and hepatitis C, particularly, interacts with alcohol (23). However, both were rare in the populations studied by us (26) and did not allow for evaluations.

Migration is a complex process that is associated with a number of health issues. Migration from Greenland to Denmark itself may influence cardiovascular risk factors (46) and cancer (47,48). We found that alcohol consumption differed according to migration, by using methods similar to those used in our comparative studies in Greenland and Denmark. In a previous investigation (4), the general consumption of alcohol was reported to be higher among Inuit in Denmark compared to Inuit in Greenland, although binge drinking was more widespread in Greenland. Although more women participated in our investigation that included other age groups, we also found that high alcohol consumption was more frequent among Inuit in Denmark compared to Inuit in the capital city Nuuk. Still, alcohol consumption in Inuit in Denmark was less frequent than among Inuit in East Greenland (Table II). This suggests that the differences may be caused by both migration and other factors. These could include differences in both the price of alcoholic drinks and income levels among Inuit in Denmark compared to those in Greenland. However, we did not include these factors in our study. Furthermore, drinking habits among Inuit in Denmark may be influenced by the generally more frequent alcohol intake common to non-Inuit (49). Still, the alcohol intake among Inuit in Denmark was markedly lower than that of non-Inuit in Denmark (49).

Alcohol intake is not fully reported by European populations, while Inuit may have the opposite attitude (3). Still, a report from Greenland found some underreporting in all parts of Greenland that did not take origin into account (50), although this reporting bias was reduced when binge drinking was taken into account. The binge drinking pattern is typical among Inuit and the associated alcohol intake was accounted for in our data on overall alcohol consumption even though we were not able to estimate the influence of binge drinking separately. Hence, Inuit in our study are not likely to underreport more prominently than non-Inuit on their alcohol consumption. Still, a limitation of our study is the lack of questions that validate the self-reported alcohol consumption.

Participants in Denmark included a 10-year increase in age range (40-to 69-year-olds) in order to increase the number of participants. This expansion in age range changed the distribution of alcohol consumption and HBV infection status among participants in Denmark by less than 2% (data not shown).

Vaccination against HBV had not been carried out in this age group in Greenland (25). Also, isolated anti-HBs has been discussed previously (25,51). To test for the potential influence of vaccination, we conducted an additional analysis. We categorized individuals that biochemically could have been vaccinated into a separate group (isolated anti-HBs). Repeated analysis, including this group, did not alter the results.

As for liver function tests, isolated elevated ALP was not seen, and any elevation was parallel to GGT elevations.

The participation rates in Denmark and in Greenland have been described previously (25,26,31). Some Greenland Inuit in Denmark have no permanent address; some non-participants are likely to be individuals with a higher alcohol intake. This may introduce selection bias in relation to alcohol intake. In addition, the categorization into alcohol group may introduce residual confounding.

Misclassification of an Inuit as non-Inuit in the study population consisting of an older age group, in 1998, is highly unlikely as members of this group were born while Greenland was kept secluded.

Higher alcohol-related mortality rates than those in other populations have previously been reported in aboriginal people (52). The low number of excess drinkers in the older age groups included in our study may be related to a number of factors including such as the costs of alcoholic beverages and spirits. Also, everyday life in the cold climate environment poses specific challenges that are more severe for the old than for the young, and may cause older persons to reduce their alcohol intake. In addition, mortality due to alcohol-related accidents in Greenland is high among at the younger age groups (9,14,15). These factors may reduce the number of Inuit with excess alcohol consumption included in our study.

Historically, Greenland has been dependent on foreign professionals and skilled workers, especially from Denmark. If the long-standing excess alcohol intake of such foreign professionals compromises their health and ability to work to the point that they can no longer live in Greenland, they may move back to Denmark. This phenomenon may reduce the number of individuals with excess alcohol intake among non-Inuit living in Greenland. Among non-Inuit in Greenland in our study, the fraction of heavy drinkers was smaller than among ethnic Danes in Denmark (49). Still, non-Inuit in Greenland in our study included fewer abstainers and more heavy drinkers compared with Inuit in the same age group. Higher income (53) and cultural differences (49) may explain this, as alcohol in Greenland is expensive (54). Over the past 25 years, the overall alcohol import to Greenland has been halved, and it is still decreasing (55).
